# Critical Role of Voltage Application Points in “Analog” and “Digital” Electrospray Ionization Mass Spectrometry

**DOI:** 10.1021/jasms.5c00082

**Published:** 2025-04-15

**Authors:** Min-Min Hung, Decibel P. Elpa, Ochir Ochirov, Pawel L. Urban

**Affiliations:** Department of Chemistry, National Tsing Hua University, 101, Section 2, Kuang-Fu Road, Hsinchu 300044, Taiwan

## Abstract

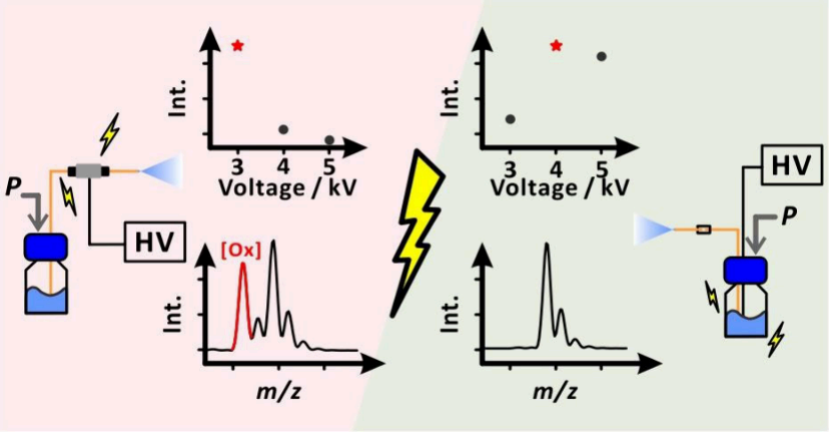

In electrospray ionization (ESI) mass spectrometry (MS), an electric DC potential is often applied to a metal capillary used to infuse a liquid sample. However, in some cases, especially when employing nanoelectrospray ionization (nanoESI), it is convenient to use a nonconducting capillary for sample delivery and spraying. In these cases, the potentials can be applied, for example, using a metal union placed in the proximity of the capillary outlet or to an electrode located in the sample reservoir near the capillary inlet. The optimum potential values, which warrant high MS signals, are different in these two operational conditions. A higher potential needs to be applied when the electrode is placed further away from the capillary outlet. Moreover, sample conductivity has a strong influence on the optimum potential values. Lower potentials must be used with highly conductive electrolytes. Thus, DC voltage scans are required to determine the optimum potentials. Applying electric potential to the electrode located in the sample reservoir, rather than metal union, significantly decreases the appearance of oxidized analyte peaks. We also show that a single-polarity square AC waveform can be applied to the union or sample reservoir electrode, and if its frequency is sufficiently high, it has a similar effect as decreasing DC voltage, allowing for digital control of electrospray with square waves (by varying duty cycle). Interestingly, the liquid meniscus oscillation frequency is independent of the AC signal frequency if the frequency is sufficiently high. Applying the AC signal in certain conditions stabilizes the electrospray plume. These observations reveal the resemblance of the ESI sample line to an *RC* circuit.

## Introduction

Electrospray ionization (ESI) is an extensively employed soft ion source for mass spectrometry (MS).^1,2^ ESI-MS is widely used to analyze a broad range of analytes, from small molecules to biological macromolecules, including metabolites, peptides, proteins, and synthetic polymers.^1,2^ It is particularly suitable for analysis of nonvolatile and thermally labile analytes, which may be challenging to analyze using other techniques.^3^ ESI-MS operates under atmospheric pressure, making it suitable for coupling with liquid chromatography (LC). Furthermore, its efficient ionization process provides high sensitivity in the analysis of samples.^3^

In conventional ESI, a direct current (DC) voltage is typically applied to the metal capillary sample emitter to facilitate the ionization process. A grounded metal union is often placed upstream of the capillary for safety reasons.^4^ Moreover, this configuration also creates a separate electrical loop that induces the electrochemical reaction in the solution, potentially affecting the analytes.^4^ The potential differences between the emitter and the grounded counter electrode (e.g., cover plate of the MS) drive the spray process.^2^ However, in some cases, especially when employing a miniaturized version of ESI source, it is convenient to use a nonconducting capillary for sample delivery and spraying. In these cases, the potentials can be applied, for example, using a metal union placed in the proximity of the capillary outlet,^5−8^ to an electrode located in the capillary emitter,^9,10^ or the sample reservoir at the capillary inlet.^11^ However, these different electrode arrangements come with their pros and cons. For instance, using a metal emitter or metal electrode induces electrochemical reactions between the solution and the metal surface due to the applied voltage.^4,9^ These reactions can lead to the oxidation or reduction of analyte and solvent or corrosion of the metal surface,^9^ potentially generating byproducts that complicate the analysis and impact the accuracy of the results.

Some studies have explored the application of different voltage waveforms, such as sine and square waves, to influence electrospray performance.^12−18^ Pulsed ESI typically relies on square wave high voltage (HV) applied to effect ionization.^12,17,19,20^ Low-frequency pulsed ESI operates at 1–100 Hz, while high-frequency pulsed ESI is generally achieved at ∼1000 Hz or higher frequencies.^13^ Pulsed ESI has several notable advantages, including reduced sample consumption,^12^ better control over the extent of analyte electrolysis during the ionization process,^15^ reduced sodium adduction and aggregation of analytes,^17^ improved signal-to-noise ratio, and lower limits of detection.^19^ Apart from pulsed ESI, another technique, alternating current (AC) ESI, typically utilizes sinusoidal high-frequency voltage to enhance ESI performance.^14,16,18^ AC ESI has some advantages including reduced ion suppression^14^ and decreased impact of changes in the acidity of buffer solvents on protein conformation.^18^

ESI-MS has found extensive applications in biomolecular analysis, particularly in the study of proteins and peptides. In the case of such species, ESI produces multiply charged ions, resulting in charge state distributions (CSDs).^1,21^ Environmental conditions such as pH, solvent composition, salt concentration, and temperature can affect protein folding and unfolding.^22,23^ Buffers or other additives are frequently employed in protein analysis to stabilize and modify protein structure, and enhance analytical performance.^1,24−27^ ESI solution additives can alter its conductivity, thus influencing spray current,^6^ and affecting the optimum electrospray voltage for gradient elution LC-MS.^28^

The present study focuses on the influence of the point of voltage application and sample conductivity on the performance of ESI with a nonconducting emitter. The influence of the point of voltage application on spray and ion current was previously reported.^5,6^ Here, we systematically compare two voltage application methods, applying high voltage to the metal union and to the sample solution vial, to explain their impact on electrospray stability, MS signal intensity, and oxidation effects. Furthermore, we explore using single-polarity square AC waveforms in ESI and demonstrate its functional equivalence to analog DC voltage control, providing new insights into the quasi-digital modulation of electrospray conditions. Given their significance in biomedical research, proteins and peptides were selected as representative analytes to evaluate the effects of such electrospray modulation based on the point of voltage application. Finally, we employed imaging techniques as diagnostic tools to visualize the electrospray process, allowing us to directly assess how AC frequency influences spray stability and Taylor cone oscillations. In this study, we employed high flow rate nanoESI (>100 nL min^–1^), which retains some of the advantages of low flow rate nanoESI (<100 nL min^–1^) while avoiding drawbacks such as the need for ultrathin tapered emitters prone to clogging.

## Experimental Section

### Chemicals

Methanol (LC-MS grade) was purchased from Merck (Darmstadt, Germany). Water (LC-MS grade) was purchased from Fisher Scientific (Waltham, MA, USA). Acetic acid was purchased from Honeywell (Charlotte, NC, USA). Ammonium acetate (98%, for HPLC) and cytochrome *c* (90%, from horse heart muscle) were purchased from Acros Organics (Geel, Belgium). Myoglobin (95–100%, from equine skeletal muscle, salt-free, lyophilized powder) and reserpine (crystallized, ≥99%, for HPLC) were purchased from Sigma-Aldrich (St. Louis, MO). Synthetic peptides [composed of three types of amino acids: histidine (H), proline (P), phenylalanine (F), and with sequences HPF, HPFHPFHPFHPFHPFHPFHPF, and HPFHPFHPFHPFHPFHPFHPFHPFHPFHPF] were custom-synthesized by BioAb (New Taipei City, Taiwan).

### NanoESI-MS Setup

**Figure 1:**
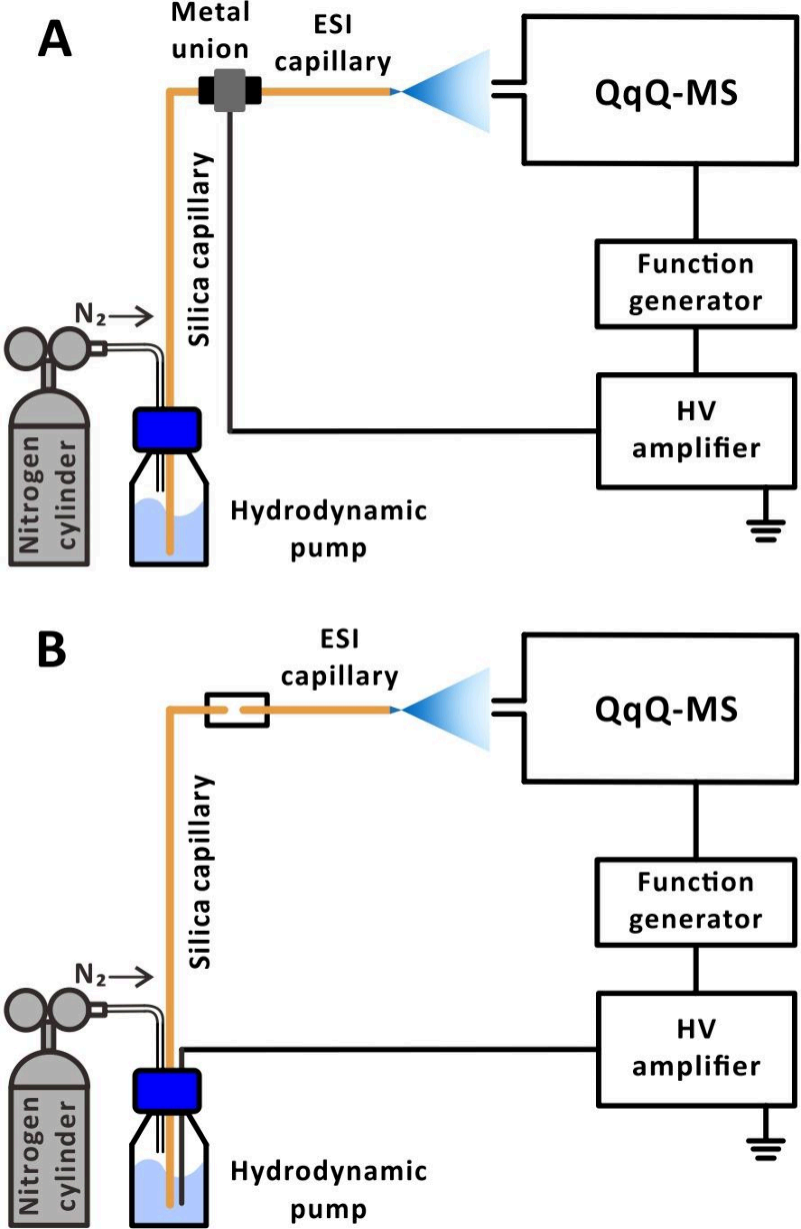
Schematic diagram of the online experimental setup: (A) voltage applied to the metal union; (B) voltage applied to the sample solution vial.

A home-built ESI setup (Figure 1) was used in the present study. A fused silica capillary (length, 6 cm; i.d., 0.050 mm; o.d., 0.375 mm; GL Science, Tokyo, Japan), which acts as an ESI emitter, was secured using a union (P-720; material, PEEK; IDEX Health & Science, Lake Forest, IL, USA) and two nuts (F-124H; material, PEEK; IDEX Health & Science). The union was used to hold the capillary in place by passing the capillary through the union and tightening the nuts to prevent sagging or displacement of the capillary during operation. The emitter assembly, including the PEEK union, was then positioned in a custom-designed 3D-printed holder. For online analysis, the emitter tip was placed ∼5 mm away from the sampling cone of the mass spectrometer. The sample solution was delivered to the emitter via a fused silica capillary (length, 60 cm; i.d., 0.050 mm; o.d., 0.375 mm; GL Science) under nitrogen gas pressure maintained at 30 kPa (Figure S1; for the relationship between the applied pressure and flow rate, see Figure S2). Notably, hydrodynamic pumps provide stable flow rates without pulsations, which is especially important for operating nanoESI-MS setups. Two different methods were employed to apply the voltage to generate the electrospray. In the first method, the HV was applied to a metal union (UH-436; through hole, 0.15 mm; material, stainless steel; IDEX Health & Science) via an HV wire connected to the HV amplifier (voltage gain: 1000 V/V, PD05034-L; Trek, Denver, CO, USA). One side of the metal union was connected to fused silica capillary linked to the hydrodynamic pump for sample delivery, while the other side was directly attached to the ESI emitter, where the liquid passed through the metal union to become charged (Figure 1A). In the second method, the HV was directly applied to the sample solution using a platinum wire, which charged the liquid as it was delivered to the emitter (Figure 1B). In this case, the metal union was replaced by a section of polytetrafluoroethylene tubing (length, 1.5 cm; i.d., 0.3 mm; o.d., 1.59 mm; part no. 58702; Supelco, Merck, Darmstadt, Germany) for delivering sample solutions by the hydrodynamic pump. In both methods, a computer-controlled function generator (Analog Discovery 2, AD2, part no. 210-321; Digilent, Pullman, WA, USA) was used to produce signals and trigger the mass spectrometer (Figure S3). In some experiments, a square waveform (frequency ranging from 1 to 25 kHz; duty cycle, 50%, unless noted otherwise) was generated using JavaScript code. The signal was transmitted to the HV amplifier to produce HV ranging from 1 to 5 kV (i.e., peak-to-peak, 4 kV; bias, 3 kV). Although some deterioration of the square shape occurred due to the rise time being in the microsecond range, the signal still presented pulsating characteristics. A relay board (model no. MTARDREL2; Kinsten, Hsinchu City, Taiwan) was powered with 5 V from the Arduino Uno microcontroller board (part no. 117895; Centenary Materials, Hsinchu City, Taiwan), and connected to the function generator to trigger the mass spectrometer to start acquisition.

### Mass Spectrometry

A triple quadrupole (QqQ) mass spectrometer (LCMS-8030, Shimadzu, Kyoto, Japan) was used for online experiments. All analyses were conducted in full scan mode. The drying gas (nitrogen) was off, while the desolvation line and the heat block temperatures were 250 and 400 °C, respectively.

### MS Data Processing

The extracted ion currents (EICs) for selected ions were exported to ASCII files from the LabSolution software (version 5.97; Shimadzu) and subsequently imported into OriginPro (version Origin 2025 (10.2); OriginLab, Northampton, MA, USA) for data processing. The average signal intensity and standard deviation were calculated for each voltage frequency region by averaging the signal.

The oxidation level for reserpine was calculated based on the following equation:
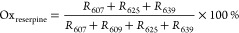
1where *R*_607_, *R*_625_, and *R*_639_ represent the main product peaks of oxidized reserpine^29^ and *R*_609_ represents the peak corresponding to reserpine.

Additional experimental details are included in the Supporting Information.

## Results and Discussion

### Comparison of Optimum Voltages for Different Points of Voltage Application

**Figure 2:**
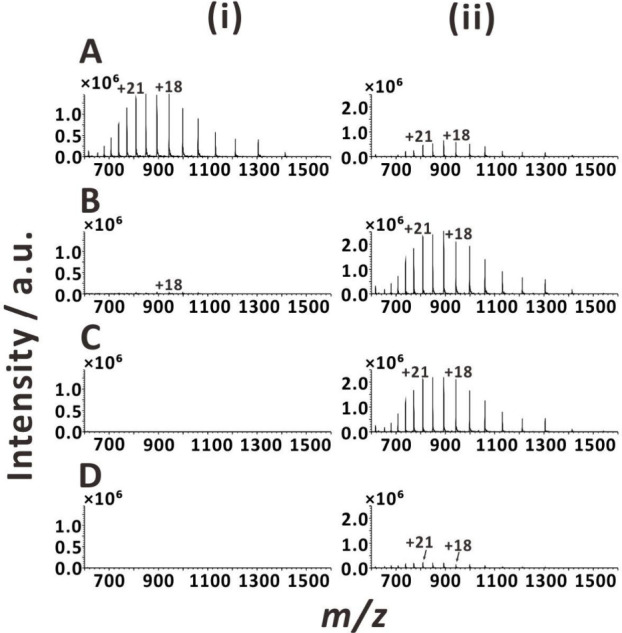
ESI mass spectra of myoglobin obtained with two DC voltage application methods: (i) voltage applied to the metal union; (ii) voltage applied to the sample solution vial. DC voltages: (A) 3 kV; (B) 4 kV; (C) 5 kV; (D) 6 kV. Sample solution: 10 μM myoglobin in 25% (v/v) aqueous methanol solution with 1% acetic acid and 1 mM ammonium acetate.


In order to verify the influence of points of voltage application on the ESI-MS performance, we compared two ESI variants: with voltage applied to the metal union close to the electrospray emitter (Figure 1A) and with voltage applied to the sample solution at the inlet of the sample line (Figure 1B). It is striking that the MS signals of the test proteins (myoglobin and cytochrome *c*) are highest at 3 kV when the DC voltage is applied to the metal union (Figures 2i and S4i), and at 4 kV when the DC voltage is applied to the sample solution vial (Figures 2ii and S4ii). This observation suggests that the electrolyte in the nonconducting capillary tubing has significant resistance, and a higher voltage is needed to achieve stable ESI when the electrode is far from the ESI emitter. The result can be explained with the dependency provided by Jackson and Enke for nonconducting needles:^6^

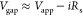
2where *V*_gap_ is the voltage across the gap, *V*_app_ is the power supply voltage, *i* is the current in the circuit, and *R*_s_ is the solution resistance. It was stated that for *iR*_s_ to be a significant fraction of *V*_app_, *R*_s_ has to be at least 10^10^ Ω.^6^

### Influence of Sample Electrolyte on the Optimum Voltages

**Figure 3:**
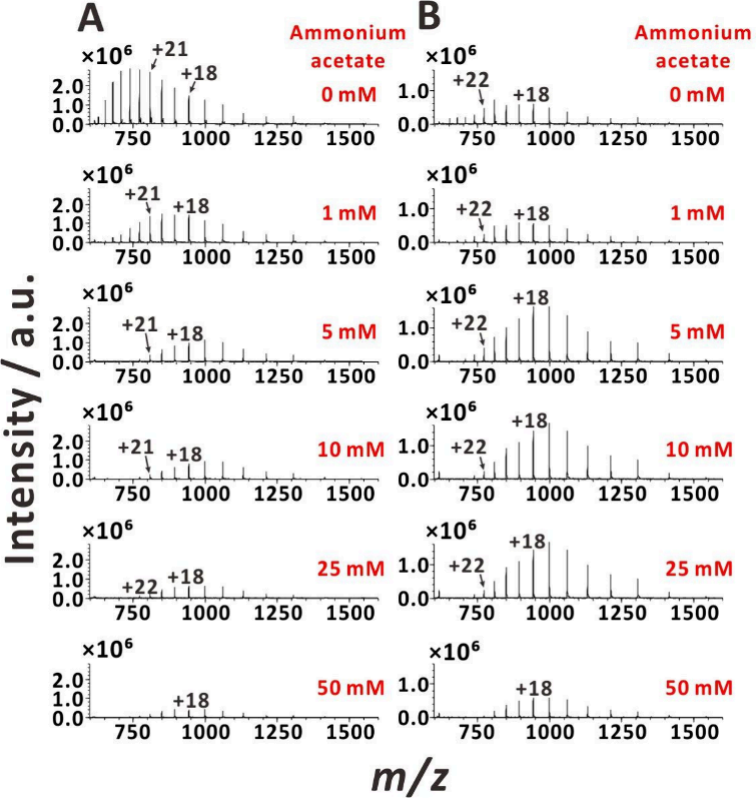
ESI mass spectra of myoglobin obtained with varying ammonium acetate concentrations under two DC voltage application methods: (A) voltage applied to the metal union; (B) voltage applied to the sample solution vial. Sample solution: 10 μM myoglobin in 25% (v/v) aqueous methanol solution with 1% acetic acid and increasing ammonium acetate concentrations (0 mM, 1 mM, 5 mM, 10 mM, 25 mM, 50 mM). DC voltage: 3 kV.

Ammonium acetate is frequently used as electrolyte in ESI-MS analyses of proteins.^24,27^ The concentrations of ammonium acetate in ESI-MS and nanoESI-MS experiments are typically in the range of 10–100 mM.^2,24^ Therefore, to further substantiate the influence of sample line resistance on the optimum ESI voltage, we acquired mass spectra of the test proteins (myoglobin and cytochrome *c*) at different concentrations of ammonium acetate while applying DC voltage (3 kV) to the metal union (Figures 3A and S5A) or to the sample solution vial (Figures 3B and S5B). As expected, the increasing concentrations of ammonium acetate caused the protein CSD to shift to lower charge states. Interestingly, when voltage was applied to the metal union, the protein signals were high at 0 mM ammonium acetate, and they decreased sharply with increasing concentrations of ammonium acetate. However, when voltage was applied to the sample solution vial, the optimum concentration of ammonium acetate was in the order of 5–25 mM. This difference can be attributed to the increased solution conductivity with higher ammonium acetate concentrations, which reduces solution resistance (*R*_s_) and alters the optimum voltage (*V*_app_) in the sample flow line made of nonconducting capillary tubing. We also investigated a narrower voltage range (2.8–3.0 kV) using myoglobin with 25 mM ammonium acetate. The results indicate that the optimum voltage for this electrolyte concentration decreases to less than 3 kV (Figure S6).

For further rationalization, we have measured the conductivities (σ) of the first sample (10 μM myoglobin in 25% (v/v) aqueous methanol solution with 1% acetic acid and varied ammonium acetate concentrations) and noted that the values ranged from 270.7 to 3013.3 μS cm^–1^ for ammonium acetate concentrations ranging from 0 to 50 mM (Table S1). Thus, with 0 mM ammonium acetate, the resistance of sample plug from the metal union to the emitter end was 1.1 × 10^9^ Ω (cf. Figure 3A, first spectrum), and the resistance of sample plug from the sample solution vial to the emitter end was 1.2 × 10^10^ Ω (i.e., exceeding the above-mentioned threshold; cf. Figure 3B, first spectrum; note: *R*_s_ = ρ*l*/*S* and ρ = 1/σ, where *S* = 1.96 × 10^–9^ m^2^). Increasing ammonium acetate concentration to 5 mM decreased the resistance of sample plug from the sample solution vial to the emitter end to 8.5 × 10^9^ Ω (cf. Figure 3B, third spectrum). Thus, the high conductivity of the sample electrolyte partly compensates for the use of long sample line and suboptimal voltage when the voltage is applied to the sample solution vial. Nonetheless, this compensation is not linear in the sense that a 10-fold increase of ammonium concentration (to 50 mM) decreases the protein signals, most likely due to ion suppression (cf. Figure 3B, sixth spectrum). Moreover, the generated currents can cause moderate Joule heating (*P* = *i*^2^*R*) in the capillary. In fact, the occurrence of Joule heating is a known phenomenon in capillary electrophoresis,^30^ and it can potentially lead to protein denaturation.^31,32^ A drawback of applying voltage to the sample solution vial is that the analyzed protein traverses the distance affected by the Joule heating (66 cm) during longer time (308 s), than it is in the case of the alternative setup with the metal union (6 cm and 28 s, respectively). Thus, the latter setup can be considered for native MS provided that the voltage is sufficiently low and the conductivity is sufficiently high, in which case the term (− *iR*_s_) in eq 2 could be neglected. However, the influence of Joule heating can be addressed by operating the two setups in a sequential manner: (1) prefilling the sample line with sample solution while the power supply is off and (2) turning on the power supply. Another way to tackle Joule heating is to implement pulsed/AC ESI instead of DC ESI.^33^

### Spray Current Measurements

Spray current measurements are performed to characterize the operation regime of electrospray.^34,35^ Here, we measured spray current for the two setups with different points of DC voltage application. When 3 kV were applied to the metal union, a pulsating nA-level current with a frequency of ∼1356 Hz was recorded (Figure S7A). The pulsations became irregular at 4 kV (Figure S7C), they disappeared at 5 and 6 kV (Figure S7E,G), and the current reached μA level. Such high currents can potentially lead to occurrence of corona discharge and change of ionization mechanism (cf. ref (36)). On the other hand, when voltages were applied to the sample solution vial, pulsations with a frequency of ∼1367 kHz were observed at 3 and 4 kV (Figure S7B,D), and they disappeared at 5 and 6 kV (Figure S7F,H). The current remained in the nA-level in this case. The recorded frequencies are lower than those reported earlier for other ESI setups.^37,38^ However, as discussed before, the pulsation frequency depends on the anchoring radius, density and surface tension of the liquid.^39^

### Influence of Point of Voltage Application on Analyte Oxidation

Electrochemical reactions involving analytes can occur in ESI-MS systems leading to artifactual peaks in the recorded mass spectra.^4,40−43^ There have been ongoing efforts to minimize the occurrence of electrochemical reaction processes, for example, by Kertesz and Van Berkel,^15,44^ Plattner et al.,^45^ Pei et al.,^46^ Lübbert and Peukert,^47^ and Han et al.^48^ Here, we verified the occurrence of reserpine oxidation in the two presented ESI-MS setups, and compared the results with those obtained using a standard ESI-MS setup with nebulizing gas.

**Figure 4:**
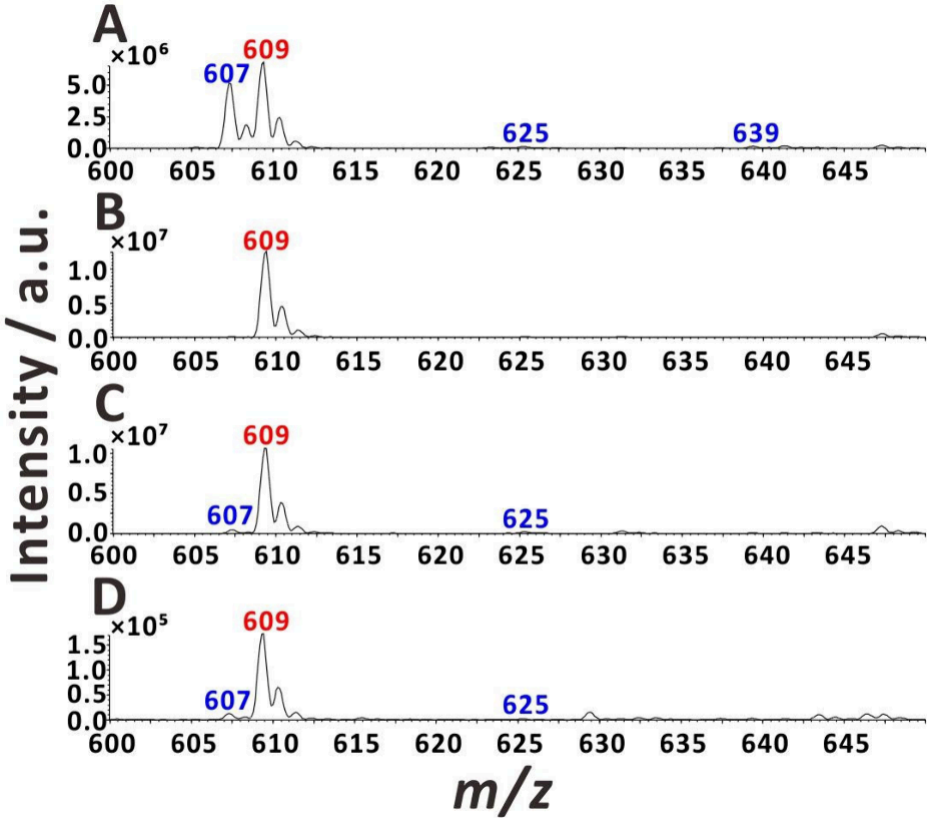
ESI mass spectra of reserpine with different DC voltage application methods: (A) voltage applied to the metal union (flow rate, ∼252 nL min^–1^; voltage, 3 kV); (B) voltage applied to the sample solution vial (1 mL; flow rate, ∼252 nL min^–1^; voltage, 5 kV); (C) voltage applied to the sample solution vial with insert (20 μL; flow rate, ∼252 nL min^–1^; voltage, 5 kV); and (D) standard ESI (flow rate, 20 μL min^–1^; voltage, 4 kV). Sample solution: 5 μM reserpine in 25% (v/v) aqueous methanol solution with 1% acetic acid.

When applying a DC voltage (3 kV) to the metal union, the main reserpine peak (*m*/*z* 609) was accompanied by a prominent oxidation product peak (*m*/*z* 607) and some other minor peaks (Figures 4A and S8). The extent of oxidation was 46.2% in that case (Table S2). Conversely, when the voltage (5 kV) was applied to the sample solution vial, the oxidation level was almost negligible (1.64%; Figure 4B). This value slightly increased when the volume of the sample solution was decreased from 1 mL down to 20 μL (5.49%; Figure 4C). Interestingly, when the standard ESI-MS setup with nebulizing gas was used (4 kV), the oxidation level was higher (8.99%; Figure 4D).

Certainly, when metal electrospray emitter is used, the oxidation reaction is more likely to occur than when using a nonconducting emitter, which is in line with the previous report.^44^ However, even when we used a metal emitter, the oxidation level was not very high because the sample flow rate was relatively high (20 μL min^–1^). Conversely, the oxidation level was very high when the sample passed through the low (nL range) dead-volume metal union at a low flow rate (∼252 nL min^–1^) due to the increased contact time with the conductive union surface biased at a HV. Nonetheless, when the voltage was supplied via a platinum wire electrode dipped in the sample solution, the observed oxidation level was very low. That is because the oxidation products accumulate near the electrode surface, which is physically separated from the capillary inlet. They may also be diluted in the relatively large sample volume (1 mL). When the sample volume is significantly decreased (20 μL) by using a vial with an insert, the distance between the wire electrode and the capillary is smaller, and the oxidation products are not diluted as much as in the previous case. In both cases, the long capillary (66 cm in total) provides sufficient separation between the sample solution vial, in which the oxidation products build up, and it takes considerable time for them to reach the emitter end, consistent with a previous report.^5^ Overall, this result shows a simple way to mitigate the influence of analyte oxidation on the mass spectrum. It also highlights the drawback of low-flow-rate ESI-MS systems with voltage supplied to the metal union. Paradoxically, this conclusion contradicts the above assertion that using the metal union setup could be advantageous for native ESI-MS.

### Apparent Voltage Reduction When Using Single-Polarity Square AC Waveform Voltage

**Figure 5:**
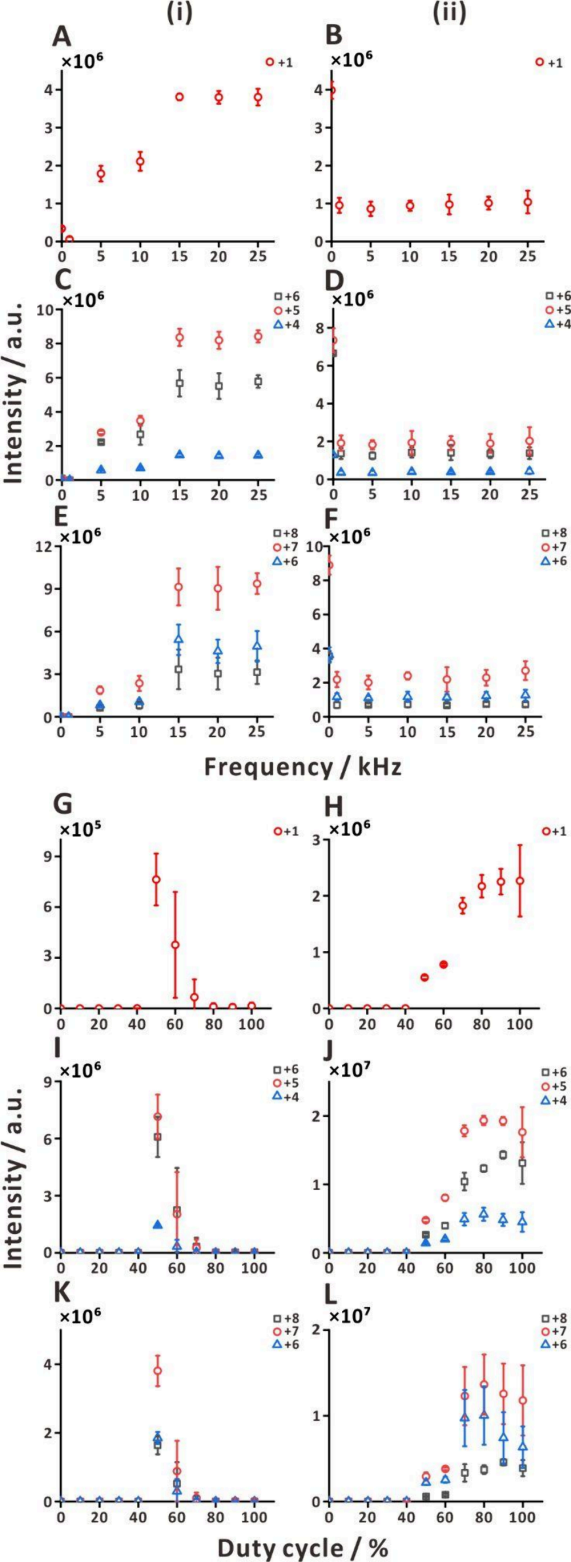
Effect of single-polarity square AC wave voltage application methods (i, ii), frequencies (A–F), and duty cycles (G–L) on signal intensity trends of HPF peptides of varying chain lengths: (i) voltage applied to the metal union; (ii) voltage applied to the sample solution vial. (A, B, G, H) Panels correspond to HPF1 (*m*/*z* 400; charge state, +1); (C, D, I, J) HPF7 (*m*/*z* 449, 539, 673; charge states, +6, +5, and +4, respectively); and (E, F, K, L) HPF10 (*m*/*z* 480, 549, 640; charge states, +8, +7, and +6, respectively). Concentration: 2 μM. Solvent: 25% (v/v) aqueous methanol solution with 1% acetic acid. Voltage span: 1–5 kV. Duty cycle in (A)–(F): 50%. Frequency in (G)–(L): 20 kHz. Sample flow rate: ∼252 nL min^–1^ (pressure, 30 kPa). The results shown in (A)–(F) and (G)–(L) were obtained on different days. Replicates, *n* = 3.

We further explored the behavior of the two ESI setups while supplying single-polarity square AC voltage (1–5 kV) instead of a DC voltage. The results were compared with those obtained for the DC voltage of 5 kV. When the AC voltage was applied to the metal union, the signals of three test peptides and two test proteins increased with the increasing frequency, and they plateaued at 15 kHz (Figures 5A,C,E and S9i). However, when the voltage was applied to the sample solution vial, the peptide signals decreased already at 1 kHz (Figures 5B,D,F and S9ii). These dependencies show that increasing AC frequency to a certain point has a similar effect as decreasing DC voltage. This is likely because at higher frequencies, more time and current is utilized for charging and discharging the electric double layer, reducing the extent of electrolysis that sustains the ESI process. The DC voltage of 5 kV is above optimum when it is applied to the metal union. The single-polarity square AC voltage (1–5 kV) effectively decreases the negative influence of this excessive voltage, when the frequency is sufficiently high. It is expected that the attenuated voltage is close to the bias voltage (3 kV) for the duty cycle 50%. In contrast, when the voltage is applied to the sample solution vial, the attenuation caused by increasing frequency leads to a decline of the signal because the attenuated voltage is below the optimum. This behavior of the ESI-MS system bears close resemblance to the characteristics of electronic low-pass filter based on *RC* circuit, which is described by the formula:^49^
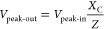
3where *V*_peak-out_ is output voltage, *V*_peak-in_ is input voltage, *X*_C_ is capacitive reactance, and *Z* is impedance. Such filters are commonly used to smooth pulse-width-modulated signals (generated by digital circuits) to create analog signals.^50^ The electric double layer can be considered as the *C* component in the *RC* circuit. It is also imaginable that, in the ESI-MS setups with nonconducting emitters, the sample electrolyte stabilizes the current due to the inertia of ions, which are periodically accelerated and decelerated by the oscillating electric field, thus enabling effective analog control. Moreover, charge separation has previously been proposed as a mechanism underlying the constant-current regulator in ESI.^6^

While analog quadrupole mass analyzers have recently been replaced by digital ones,^51−53^ the above results suggest that ESI can also be driven in a digital manner. To verify this assertion further, we have conducted an experiment, in which the two ESI-MS setups (cf. Figure 1) were operated with 20 kHz square waves (1–5 kV) with different duty cycles. That result shows that, when applying the voltage to the metal union, the signals of all test peptides are high within a narrow duty cycle range (50–60%; Figure 5G,I,K). On the other hand, when the voltage is applied to the sample solution vial, the signals increase for the duty cycles in the range 50–70%, and then reach a plateau (Figure 5H,J,L). Thus, single-polarity square AC wave duty cycle indeed enables controlling ESI source in a similar way as varying DC voltage.

### Plume Visualization in DC and AC Electrosprays

**Figure 6:**
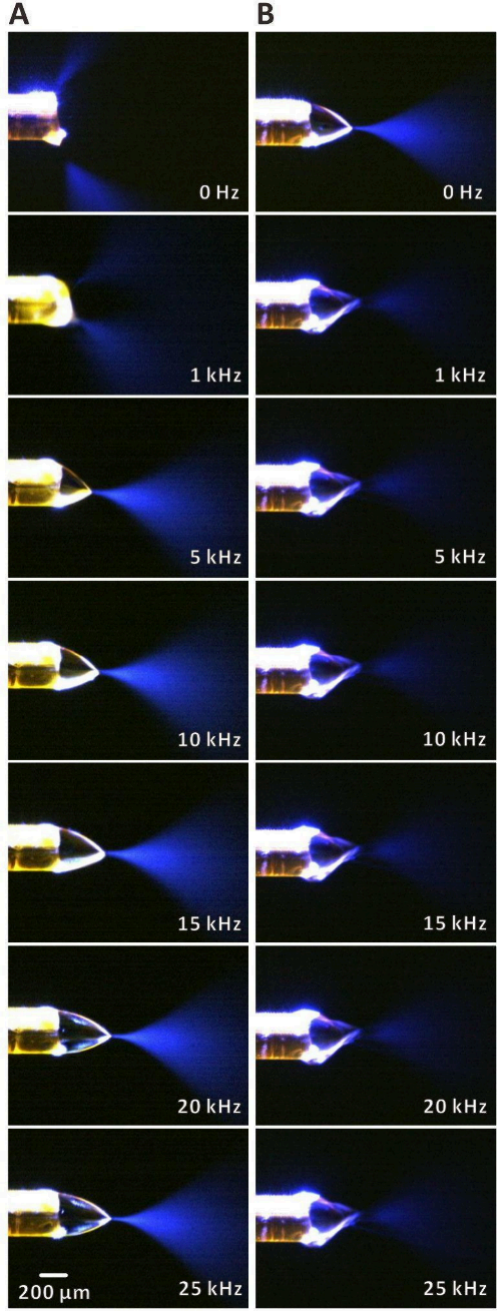
Images of electrospray plumes under different single-polarity square AC wave voltage frequencies: (A) voltage applied to the metal union; (B) voltage applied to the sample solution vial. Solution: 25% (v/v) aqueous methanol solution with 1% acetic acid. Voltage span: 1–5 kV.

We have further conducted imaging of electrospray plumes using light scattered from a laser beam (Figure 6). In the case of voltage applied to the metal union, a multijet spray was formed at 0 Hz (5 kV) and 1 kHz (1–5 kV; Figure 6A, Movie S1). The plume developed properly at higher frequencies. On the other hand, when the voltage was applied to the sample solution vial, a proper plume developed already at 0 Hz (DC), and its size diminished with the increasing frequency (Figure 6B, Movie S2). These observations are in line with the above discussion on the apparent voltage reduction caused by the application of single-polarity square AC waves to the electrospray electrode. The voltage of 5 kV is too high to sustain formation of regular plume, when it is applied to the metal union positioned close to the electrospray emitter. Increasing frequency leads to reduction of the apparent voltage and formation of high-quality plumes. When 5 kV are supplied to the electrode placed in the solution, a regular plume can be formed due to the relatively high resistance of the sample solution in the capillary. In this case, the increasing frequency leads to suboptimal attenuated voltage for electrospray plume development.

### Taylor Cone Visualization in DC and AC Electrosprays

To further verify the influence of DC and AC voltages on the electrospray process, we carried out imaging of Taylor cone using a high-speed camera (Figure S10). In all cases, the liquid was anchored on the outer diameter. When DC voltage of 5 kV was applied to the metal union, no meniscus oscillations were observed. When DC voltage was decreased to 3 kV, the meniscus oscillated near a frequency of ∼1308 Hz. For a single-polarity square AC signal (1–5 kV) at 1 kHz, the spray was unstable. When DC voltage (5 kV) was applied to the sample solution vial, the meniscus elongated, then it split, initiated a period of pulsations, and eventually returned to a stable cone-jet (astable regime, cf. ref (54)). These observations point to transitions between different electrospray regimes. For the AC (1–5 kV) frequency range 5–25 kHz, a bistable condition was observed with two meniscus oscillation frequencies: ∼957 and 1290 Hz (Figure S11). The AC frequency change did not affect the meniscus frequency.

Both imaging experiments focusing on the electrospray plume and liquid meniscus further substantiate the hypothesis that the ESI system made of nonconducting silica capillaries resembles an *RC* circuit, which is insensitive to short-term (submillisecond) variations of the driving voltage. Although further insight could potentially be brought by spray current measurements, they could not be conducted due to a high displacement current induced in the Faraday plate by the AC signal. It should be noted that the *RC* characteristic of electrospray makes it compatible with transient voltage sources such as triboelectric generators (cf. refs (55) and (56)).

## Conclusions

While ESI-MS analyses can readily be performed using nonconducting capillary emitters, the applied voltage and the application point need to be selected rationally. The optimum voltage applied to the wire electrode placed in the sample solution is higher than that applied to the metal union mounted on the capillary. When applying voltage upstream from the nanoESI emitter, it is important to note that the optimum voltage is strongly affected by the conductivity of the sample. This has implications on many experiments involving biomolecules, which are dissolved in high-conductivity electrolytes. Applying single-polarity square AC waves has a similar effect as decreasing the voltages, either decreasing or increasing MS signals. This effect enables quasi-digital control of the electrospray regime by altering duty cycle. Modulating the duty cycle provides a means of controlling the ESI process, which has implications on the ESI-MS system with intermittent voltage sources. It also reveals resemblance of the ESI sample line to an *RC* circuit. The *RC* circuit-like property of ESI affects the behavior of Taylor cone and electrospray plume positively, by averaging short-term changes in voltage. Thus, it stabilizes the operation of the ESI process. It is counterintuitive that the frequency of the natural electrospray pulsations does not synchronize with the single-polarity square AC frequency used to drive the electrospray. Applying voltage to the sample reservoir results in the dilution of oxidation products before reaching the electrospray emitter, thereby reducing their impact on analysis compared to voltage application at the metal union. This method effectively reduces oxidized analyte peaks in mass spectra, which is especially advantageous in low-flow nanoESI systems. The insights from the plume and Taylor cone visualizations reveal key differences in electrospray behavior based on the point of voltage application, providing new perspectives that were not observed in earlier ESI studies. Overall, the above results bring insights on the operation of ESI, and they can help analysts to customize ESI-MS systems depending on the emerging analytical needs regarding sample consumption, required flow rate, prevention of heat-induced denaturation, vulnerability of analytes to oxidation, electrolyte composition, and other technical constraints.

## Supporting Information Available

Additional experimental details (spray current measurements; visualization of electrospray plume; visualization of liquid meniscus pulsations at a high speed); Tables S1 and S2 listing conductivities of myoglobin samples and evaluation results of analyte oxidation; Figures S1–S12 showing scheme of hydrodynamic pump tubing connections, relationship between the applied pressure and flow rate for ESI setup, electronic control circuit, mass spectra of cytochrome *c* obtained with two DC voltage application methods, mass spectra of cytochrome *c* obtained with varying ammonium acetate concentrations under two DC voltage application methods, mass spectra of myoglobin obtained with different DC voltages applied to the metal union, DC ESI spray current measurements with two different voltage application methods, mass spectra of oxidized reserpine, effect of single-polarity square AC wave voltage application methods and frequencies, high-speed camera images revealing liquid meniscus oscillations under different conditions, relationship between the applied voltage frequency and the liquid meniscus oscillation frequency, and offline ESI imaging setups; computer codes (PDF)
Movie S1 showing ESI spray plume for voltage applied to metal union (MP4)
Movie S2 showing ESI spray plume for voltage applied to sample solution vial (MP4)
