# Supplementary material for: Critical Role of Voltage Application Points in “Analog” and “Digital” Electrospray Ionization Mass Spectrometry
Source: J Am Soc Mass Spectrom. 2025 Apr 15;36(5):1191–9. doi: 10.1021/jasms.5c00082 (PMC12063163; doi:10.1021/jasms.5c00082)
Supplement: Supplementary file 1 — js5c00082_si_001.pdf [file js5c00082_si_001.pdf]

SUPPORTING INFORMATION

Critical Role of Voltage Application Points in "Analog" and "Digital" Electrospray Ionization Mass Spectrometry

Min-Min Hung, Decibel P. Elpa, Ochir Ochirov, Pawel L. Urban*

Department of Chemistry, National Tsing Hua University

101, Section 2, Kuang-Fu Rd., Hsinchu, 300044, Taiwan

* Corresponding author:

P.L. Urban (urban@mx.nthu.edu.tw)

ADDITIONAL EXPERIMENTAL DETAILS

Spray current measurements

We measured the electric current generated by electrospray between the ESI emitter and a custom, square-shaped Faraday plate (10 mm × 10 mm × 0.8 mm). The plate consisted of an insulating board with a layer of copper foil on one side. The distance between the emitter and the plate was 5 mm. A coaxial cable with a BNC connector at one end and an exposed conductor soldered to the Faraday plate at the other end provided the connection to a transimpedance amplifier (low-noise gain: 10^6 V A^{-1}; DLPCA-200; FEMTO, Berlin, Germany). The amplifier transmitted signals to a digital oscilloscope (Analog Discovery 2; Digilent, Pullman, WA, USA). The measurements were recorded using accompanying software (WaveForms, ver. 3.23.4; Digilent, Pullman, WA, USA). Electric current measurements were performed under two conditions: the DC voltage applied to the metal union and to the sample solution, respectively.

Visualization of electrospray plume

The imaging setup was built to capture the image of the electrospray plume (**Figure S12A**). The ESI system was the same as the ESI-MS setup. A grounded counter electrode (model no. CGS-1015-0.8-Single, Centenary Materials, Hsinchu City, Taiwan) was positioned ~ 5 mm from the tip of the ESI emitter, making the condition similar to that used in MS analysis. The industrial camera (model no. DFK 33UX174; The Imaging Source, Bremen, Germany) was coupled to a stereomicroscope (model no. SMZ745T; Nikon, Tokyo, Japan), and was placed orthogonal to the ESI emitter. A laser pointer (model no. 450P-100-BL; wavelength, 405 nm; output power: < 1 mW; Jolooyo, Wuhan, China) was positioned at an angle of ~ 90° relative to the emitter to illuminate the electrospray plume. The output of the industrial camera was linked to the computer, and the images were acquired using the IC Capture software (version 1.1.0.23; The Imaging Source). The optimized imaging parameters were: exposure time, 0.67 s; frame rate, 15 fps; gain value, 37.9 dB.

Visualization of liquid meniscus pulsations at a high speed

A high-speed camera (HSC), i-SPEED 727 (iX Cameras, Rochford, UK), was utilized to capture the pulsation phenomena of the Taylor cone, with a primary focus on the cone itself (**Figure S12B**). The experimental setup was identical to the industrial camera imaging configuration, with the ESI emitter positioned 5 mm in front of the ground electrode. The HSC was equipped with teleconverters (Teleplus HD DGX 1.4x and 2.0x; Kenko, Tokyo, Japan) attached to the lens to enhance magnification. Images of the Taylor cone dynamics were recorded using the HSC, connected to a computer *via* an Ethernet cable. Image acquisition was controlled using iSPEED Control software (version 25.0.18.3; iX Cameras). The HSC was operated at a frame rate of 272,251 fps, with a 200 × 200 pixels resolution, and an exposure time of 308 ns. To achieve shadowgraph imaging, a halogen lamp (OSL2IR; Thorlabs, Newton, NJ, USA) was placed on the opposite side of the camera, illuminating the Taylor cone to create a clear contrast in the captured images. By analyzing the captured images, the difference in the

number of frames between consecutive cone appearances is determined. This difference is then multiplied by 1/(frame rate) to obtain the oscillation period. The oscillation frequency is then calculated using 1/period.

ADDITIONAL TABLES

Table S1. Conductivities of 10 µM myoglobin in 25% (v/v) aqueous methanol solution with 1% acetic acid and different ammonium acetate concentrations. Conductometer: model no. EC310F-P; REX, Shanghai, China.

Concentration / mM	Conductivity / µS cm^{-1}
0	270.7 ± 7.0
1	254.3 ± 6.7
5	395.7 ± 6.7
10	682.7 ± 17.0
25	1575.7 ± 10.4
50	3013.3 ± 15.3

Table S2. Evaluation of analyte oxidation. Sample solution: 5 μM reserpine in 25% (v/v) aqueous methanol solution with 1% acetic acid. Voltage applied to the metal union: flow rate, ~ 252 nL min^{-1}; voltage: 3 kV. Voltage applied to the sample solution vial (1 mL): flow rate, ~ 252 nL min^{-1}; voltage: 5 kV. Voltage applied to the sample solution vial with insert (20 μL): flow rate, ~ 252 nL min^{-1}; voltage, 5 kV. Standard ESI: flow rate, 20 μL min^{-1}; voltage, 4 kV.

	**Voltage applied to the metal union**	**Voltage applied to the solution (1 mL)**	**Voltage applied to the solution (20 μL)**	**Standard ESI**
$Ox_{reserpine}$ (%)	46.2 ± 2.31	1.64 ± 0.35	5.49 ± 0.56	8.99 ± 1.22

ADDITIONAL FIGURES

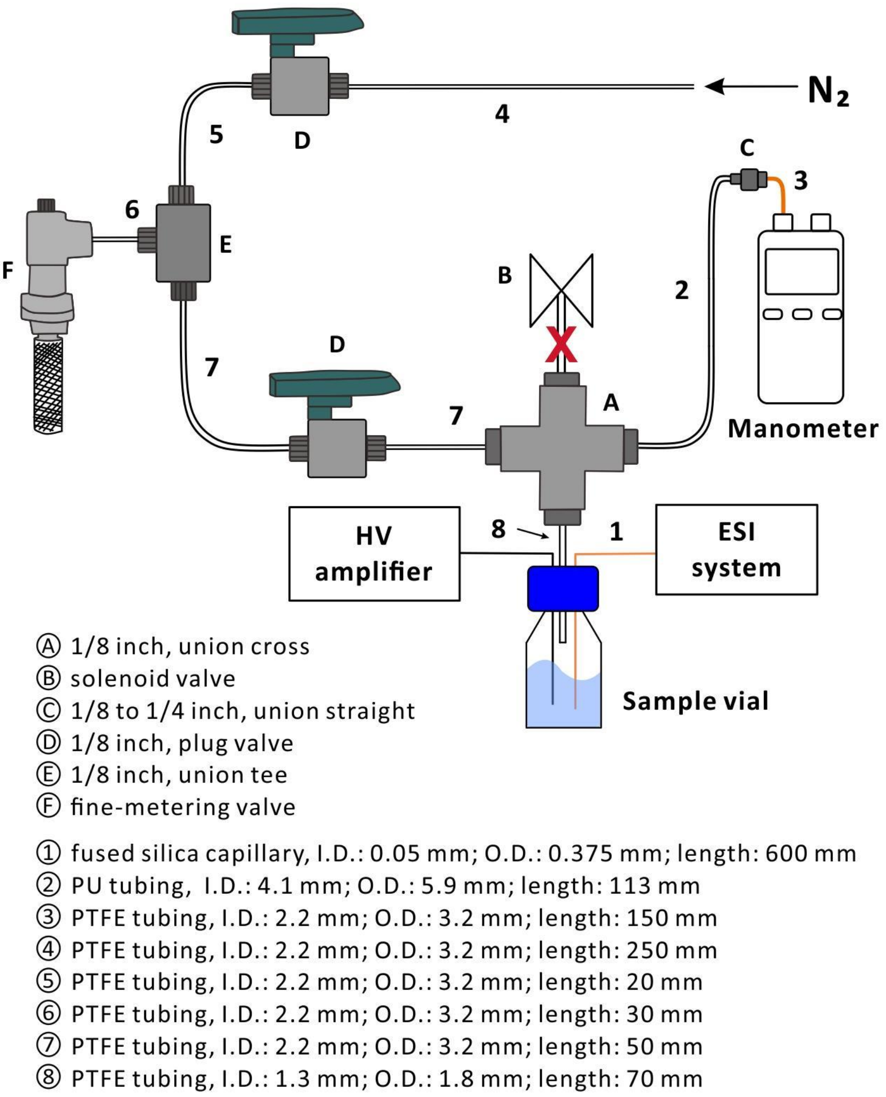

Figure S1. The scheme of tubing connections in the hydrodynamic pump (not drawn to scale). PU – polyurethane; PTFE – polytetrafluoroethylene.

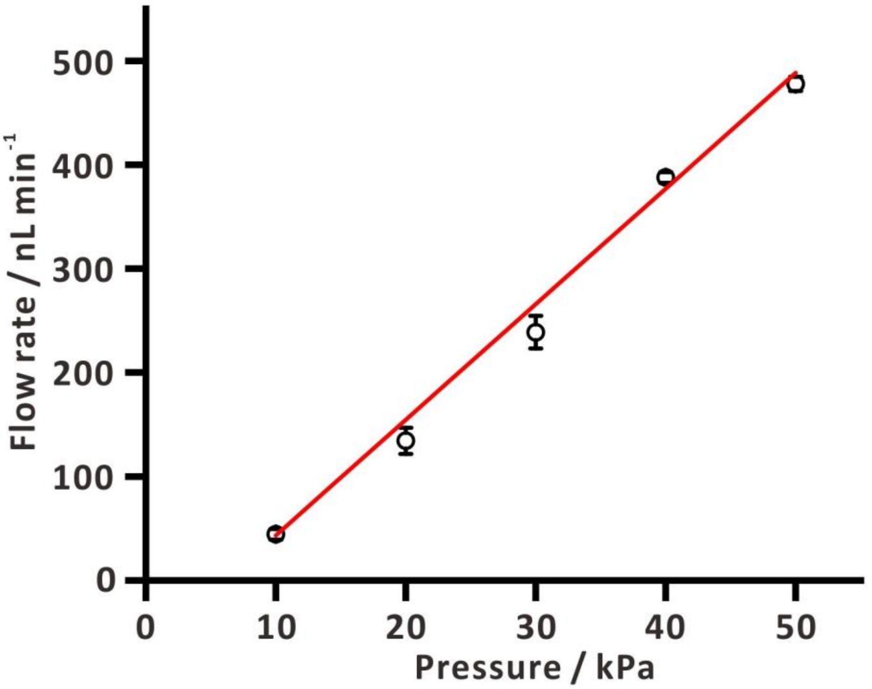

Figure S2. The relationship between the applied pressure and flow rate for ESI setup (ESI capillary I.D: 50 μm). The total capillary length was 66 cm, consisting of a 60 cm fused silica capillary connected to the hydrodynamic pump, with a PTFE tubing serving as the connection between the pump and the 6 cm ESI emitter. No voltage was applied to the system. The solution was 25% (v/v) aqueous methanol solution with 1% acetic acid (pH 2.6). Calibration equation: *Flow rate* = (11.1 $\pm$ 0.4) *Pressure* + (-68.1 $\pm$ 12.9), R^2 = 0.995. Replicates, n = 3.

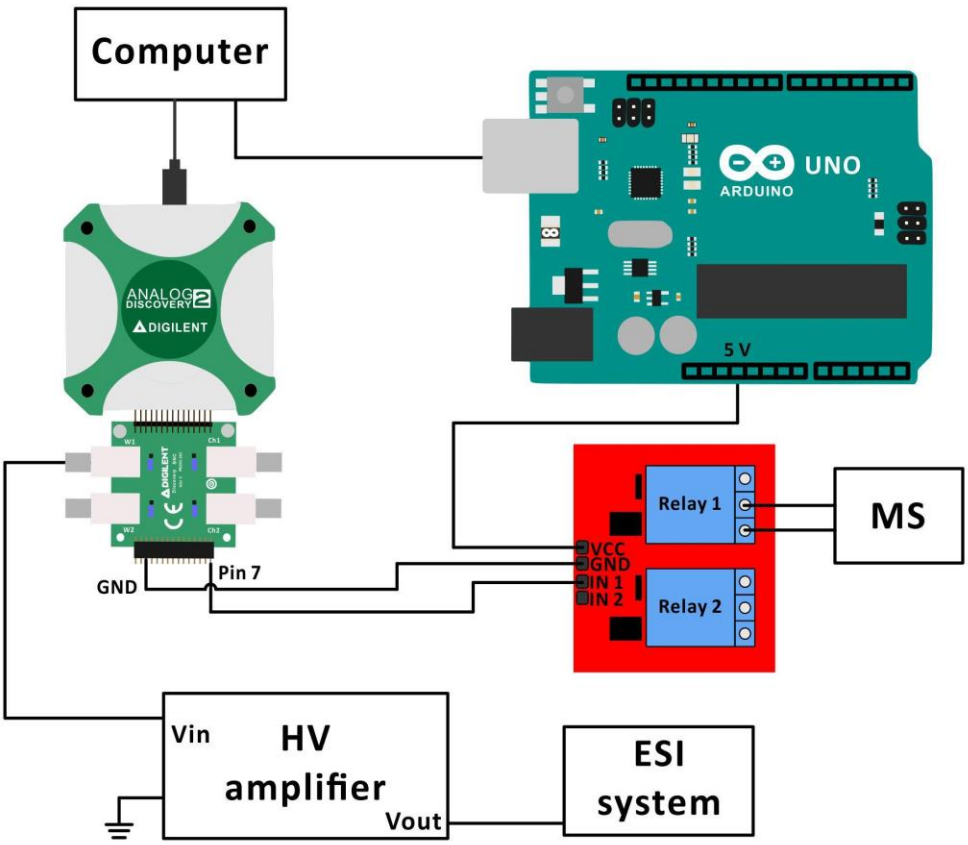

Figure S3. Simplified scheme of the electronic circuit used to control the HV signal and trigger MS. Note that the Arduino Uno board supplies 5 V to the relay board, so that one does not need to use an additional power supply.

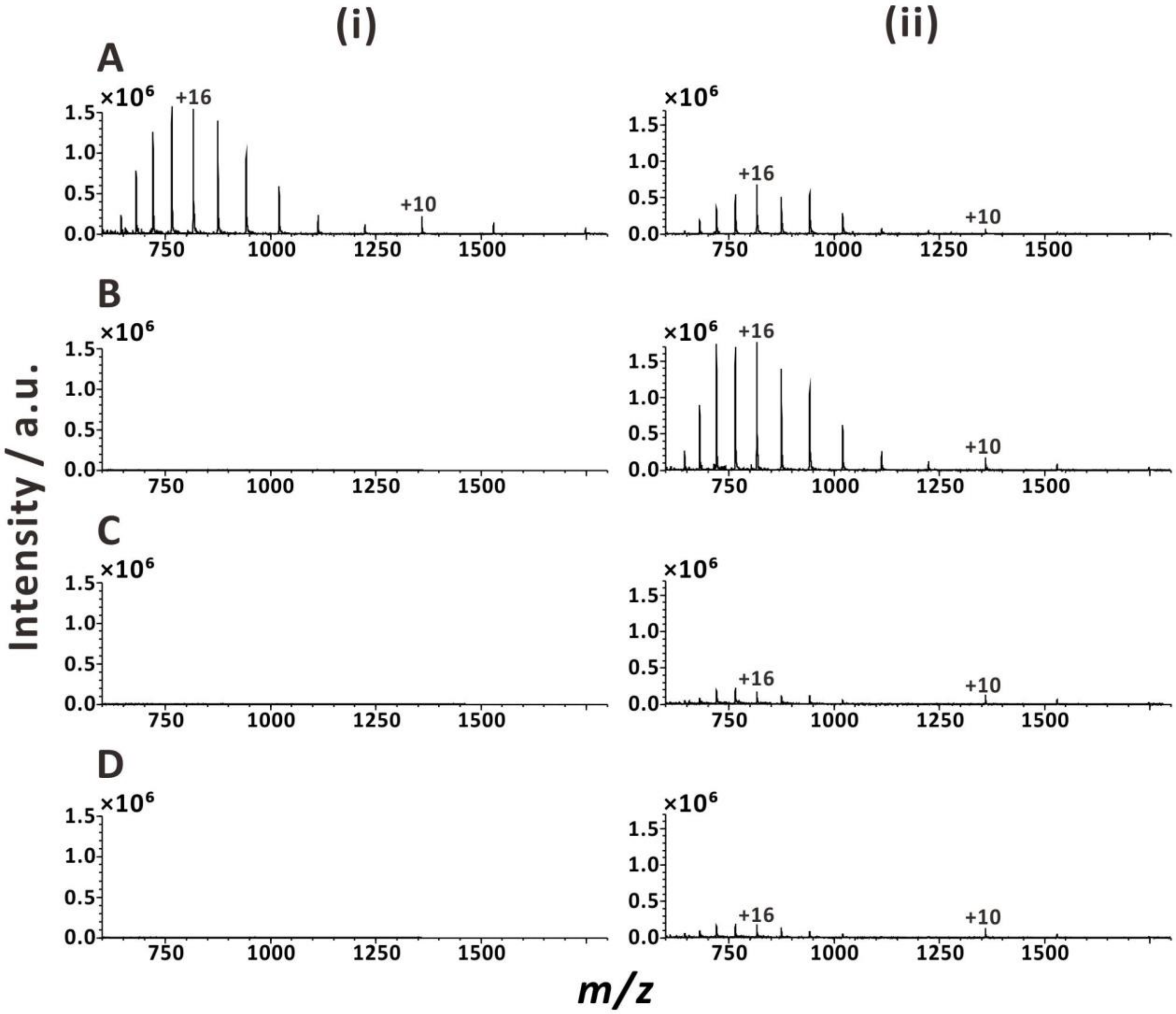

Figure S4. ESI mass spectra of cytochrome *c* obtained with two DC voltage application methods: (i) voltage applied to the metal union; (ii) voltage applied to the sample solution vial. DC voltages: (A) 3 kV; (B) 4 kV; (C) 5 kV; (D) 6 kV. Sample solution: 10 μM cytochrome *c* in 25% (v/v) aqueous methanol solution with 1% acetic acid and 1 mM ammonium acetate.

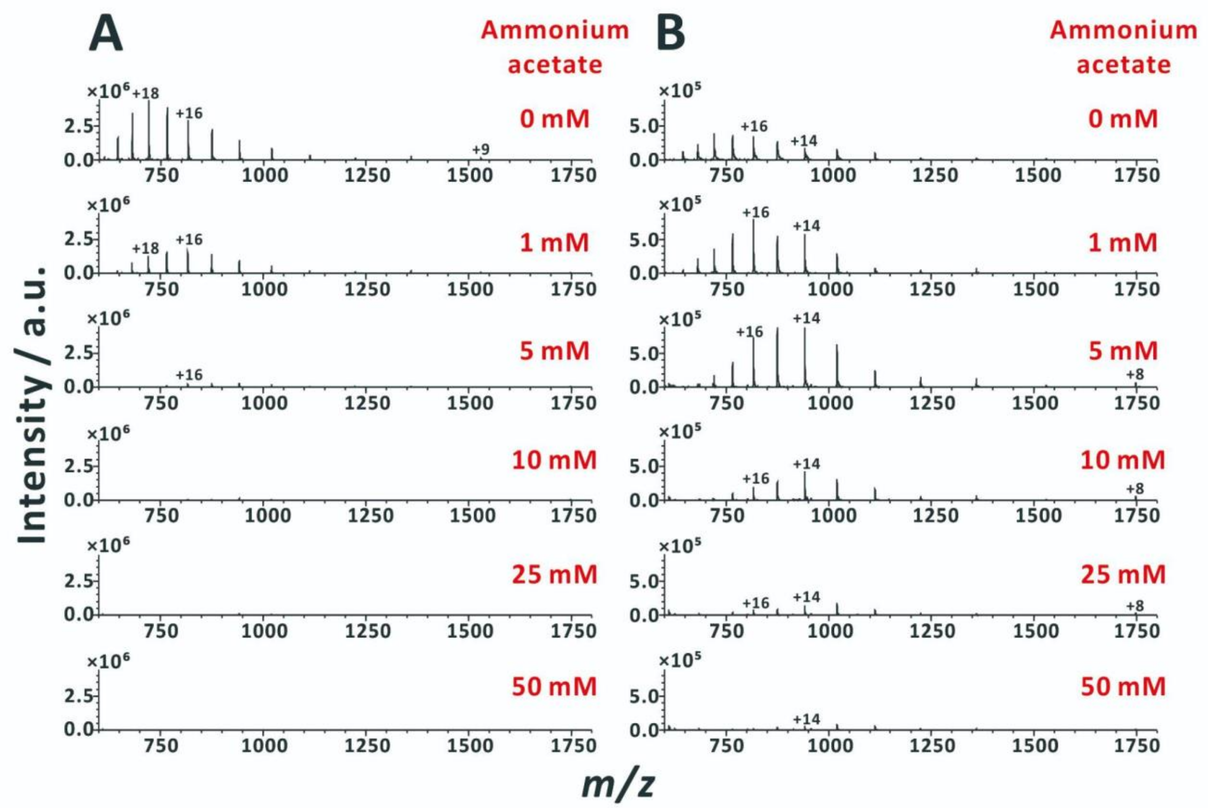

Figure S5. ESI mass spectra of cytochrome *c* obtained with varying ammonium acetate concentrations under two DC voltage application methods: (A) voltage applied to the metal union; (B) voltage applied to the sample solution vial. Sample solution: 10 μM cytochrome *c* in 25% (v/v) aqueous methanol solution with 1% acetic acid and increasing ammonium acetate concentrations (0 mM, 1 mM, 5 mM, 10 mM, 25 mM, 50 mM). DC voltage: 3 kV.

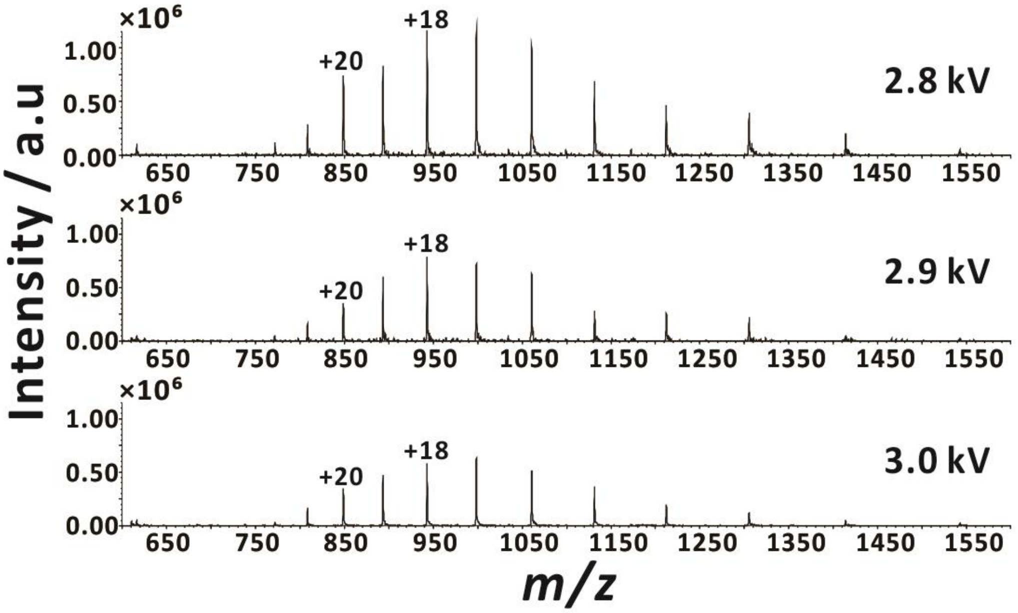

Figure S6. ESI mass spectra of 10 μM myoglobin in 25% (v/v) aqueous methanol solution with 1% acetic acid and 25 mM ammonium acetate, obtained with different DC voltages applied to the metal union.

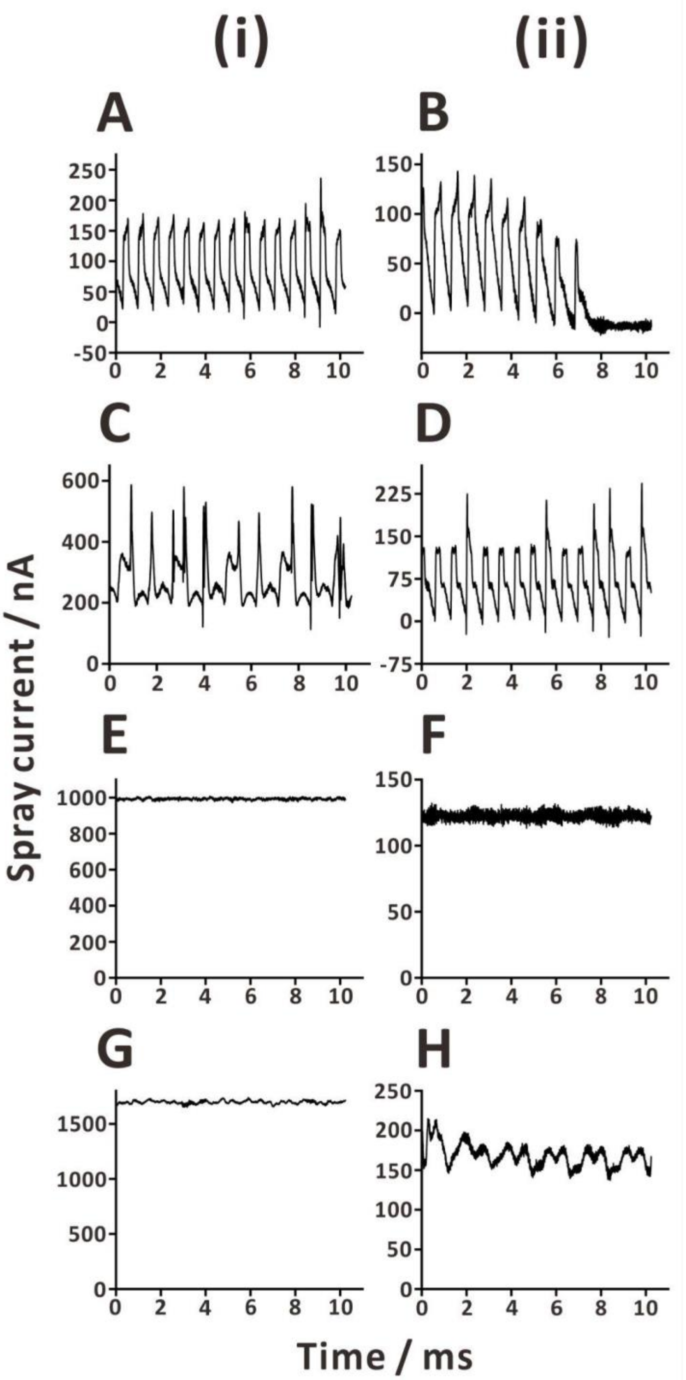

Figure S7. DC ESI spray current measurements with two different voltage application methods: (i) voltage applied to the metal union; and (ii) voltage applied to the sample solution vial. Voltages: (A,B) 3 kV; (C,D) 4 kV; (E,F) 5 kV; (G,H) 6 kV. Solution: 25% (v/v) aqueous methanol solution with 1% acetic acid. Data smoothing was performed using the Savitzky-Golay filter with a 50-point window (2nd order).

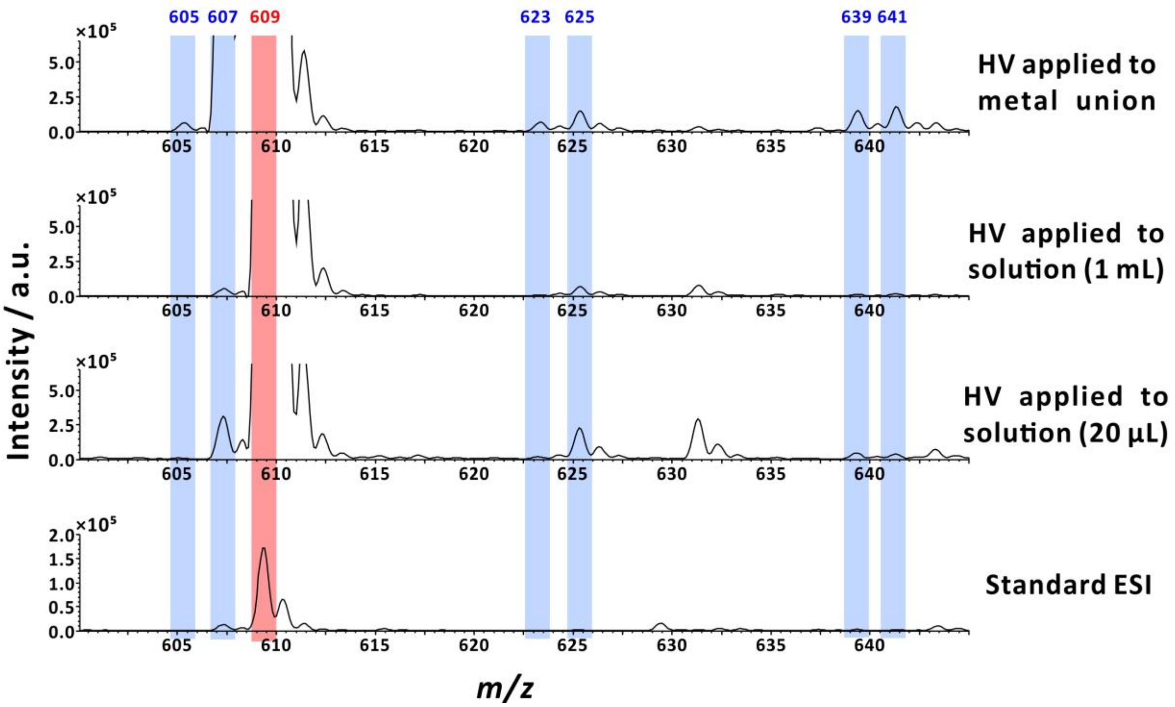

Figure S8. ESI mass spectra of 5 µM reserpine in 25% (v/v) aqueous methanol solution with 1% acetic acid obtained with different voltage application methods. Unoxidized reserpine peak highlighted in red (*m/z* 609), and oxidized peaks highlighted in blue (*m/z* 605, 607, 623, 625, 639, and 641).

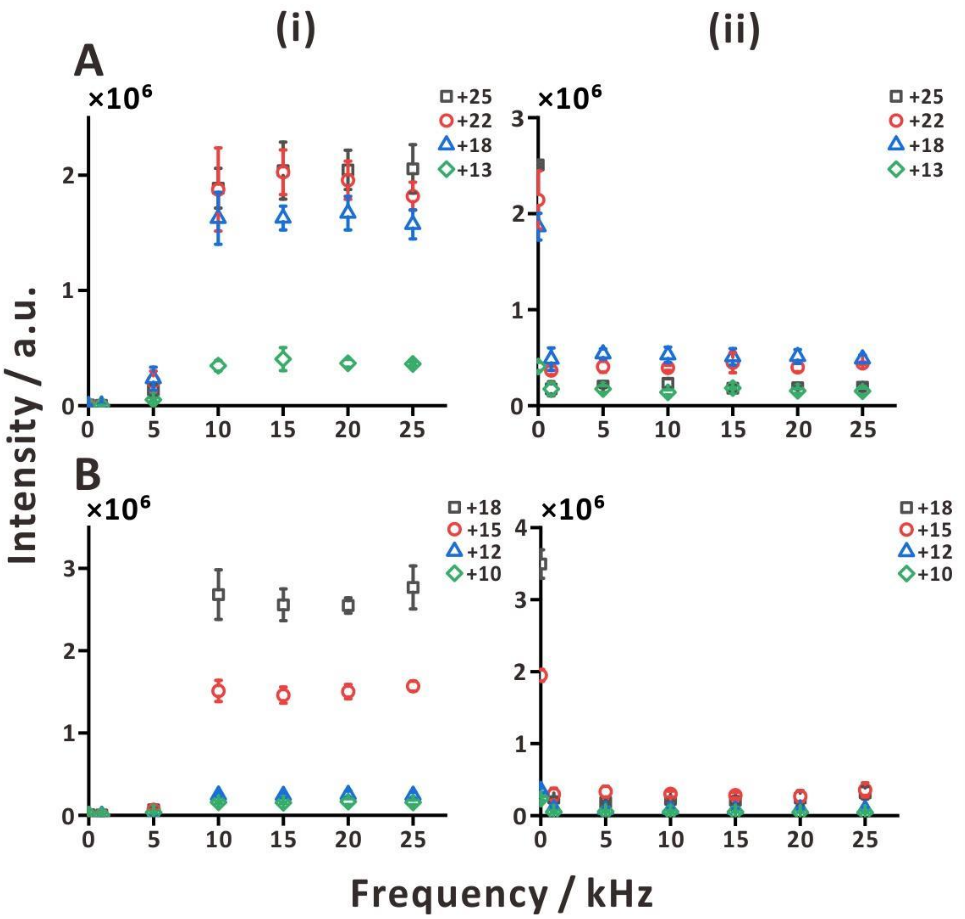

Figure S9. Effect of single-polarity square AC wave voltage application methods and frequencies on signal intensity trends of two proteins: (i) voltage applied to the metal union; and (ii) voltage applied to the sample solution vial. Panels (A) correspond to myoglobin; and (B) to cytochrome *c*. Concentration: 10 μM. Solvent: 25 % (v/v) aqueous methanol solution with 1% acetic acid. Voltage span: 1-5 kV. Frequencies increased stepwise: 0, 1, 5, 10, 15, 20, and 25 kHz. Sample flow rate: ~ 252 nL min^{-1} (pressure: 30 kPa). Replicates, *n* = 3.

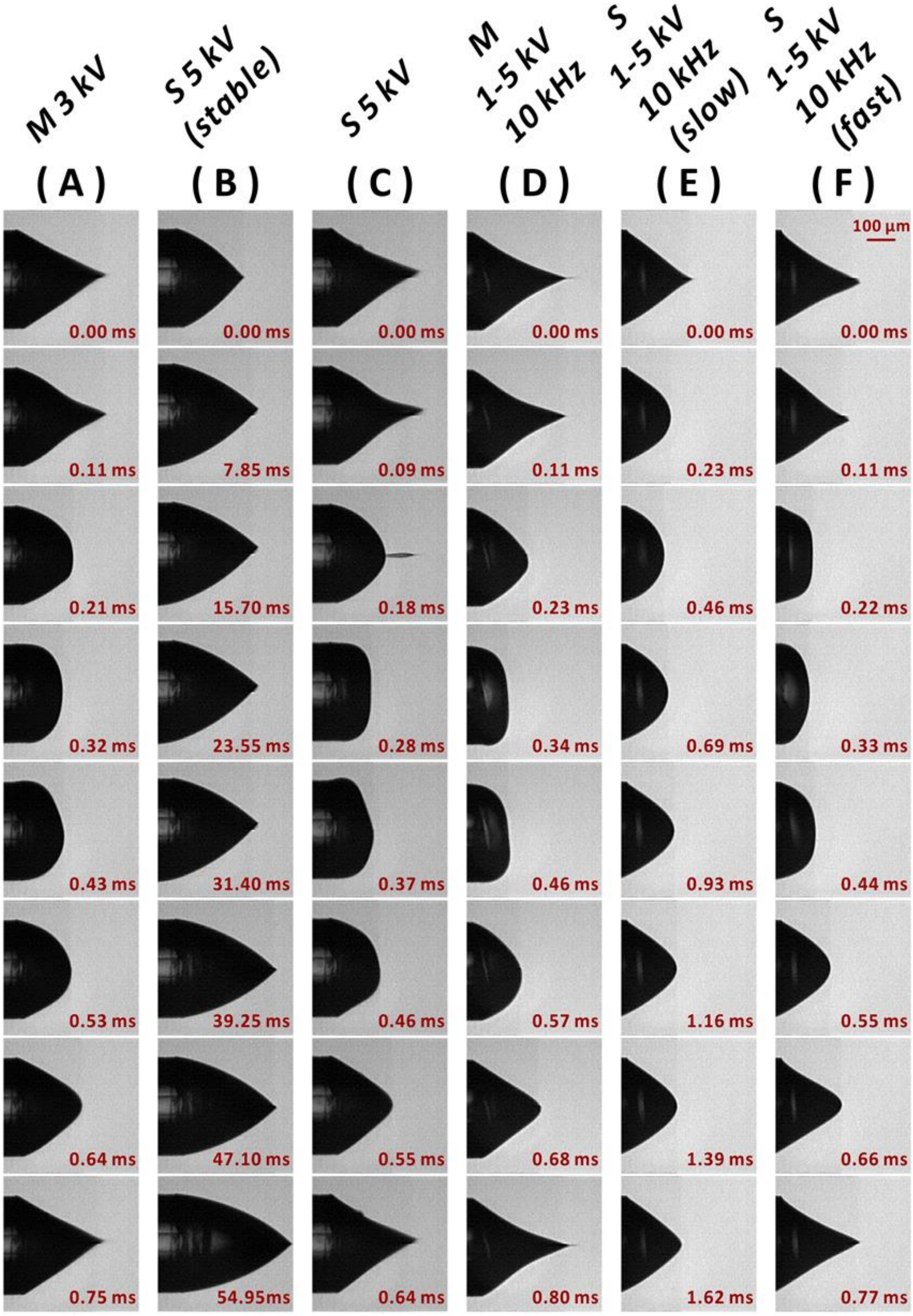

Figure S10. HSC images revealing liquid meniscus oscillations under different conditions. Solution: 25% (v/v) aqueous methanol solution with 1% acetic acid. M represents the experiment in which voltage was applied to the metal union, S represents the experiment in which voltage was applied to the sample solution vial. (A) DC 3 kV; (B) DC 5 kV, stable cone-jet; (C) DC 5 kV, pulsating cone jet; (D) AC 1-5 kV; (E) AC 1-5 kV, slow pulsation; (F) AC 1-5 kV, fast pulsation.

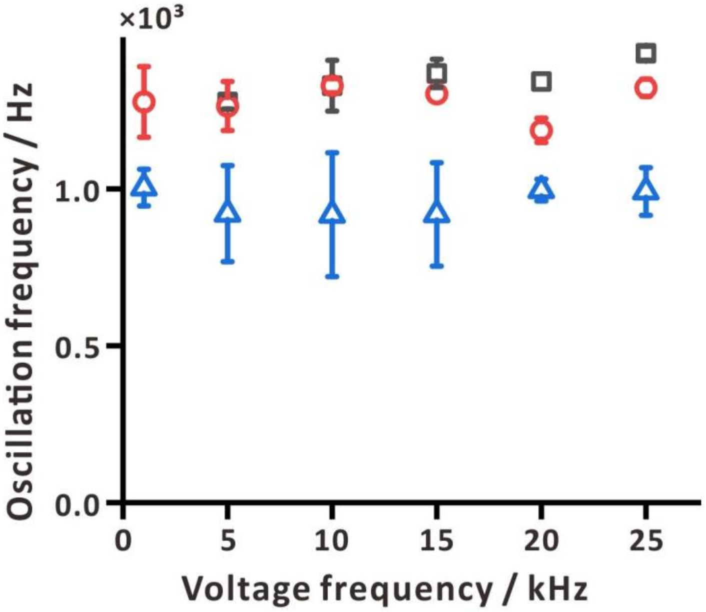

Figure S11. Relationship between the applied voltage frequency and the liquid meniscus oscillation frequency. Solution: 25% (v/v) aqueous methanol solution with 1% acetic acid. Voltage span: 1-5 kV. Grey hollow squares represent the experiment in which voltage was applied to the metal union. Blue hollow triangles represent experiments with voltage applied to the sample solution vial, resulting in lower Taylor cone oscillation frequencies. Red hollow circles represent experiments in which voltage was applied directly to the sample solution vial, resulting in higher Taylor cone oscillation frequencies.

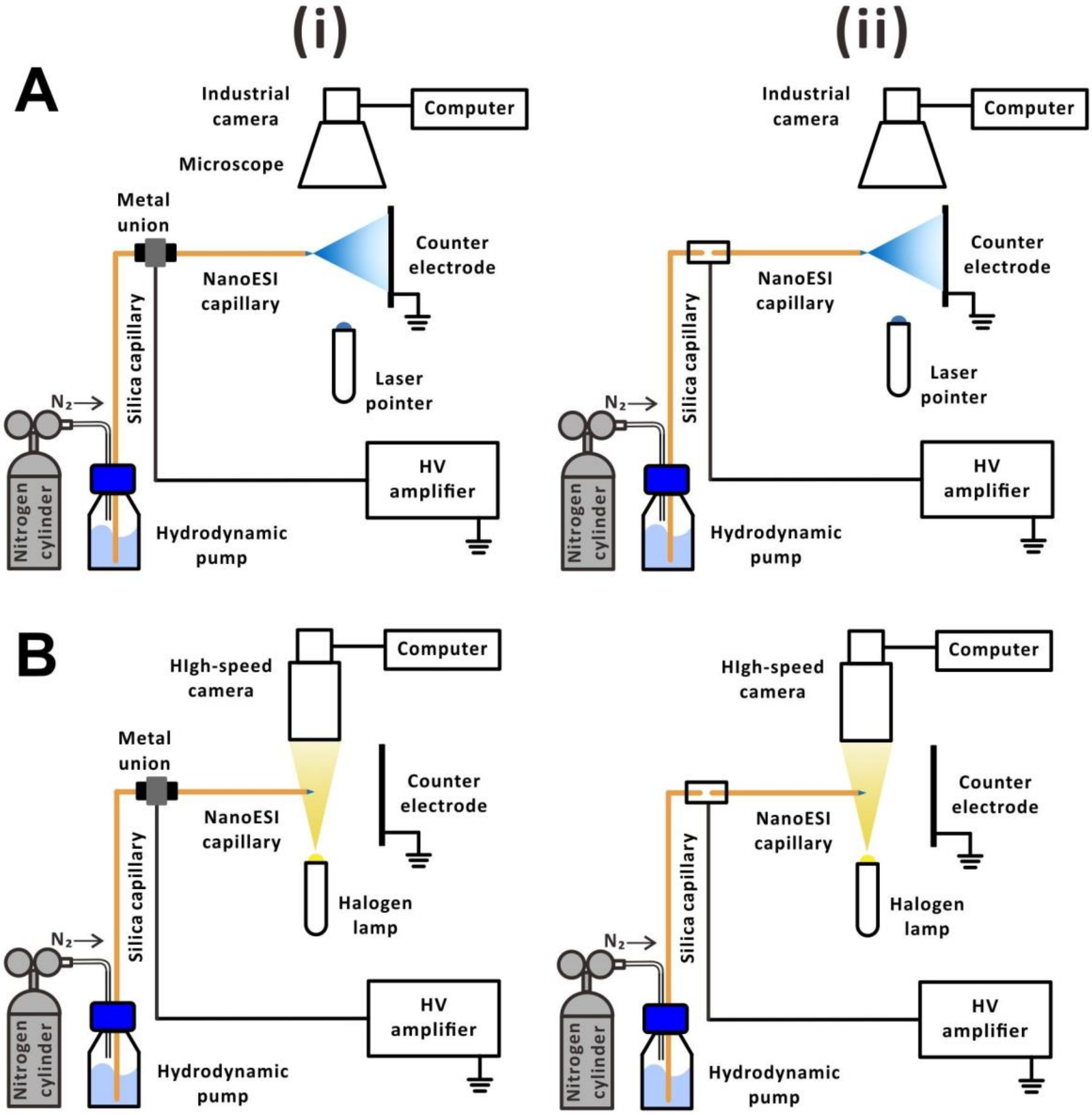

Figure S12. Offline ESI setups for imaging: (A) configuration with an industrial camera; and (B) configuration with a high-speed camera. Each setup has two variants for two different voltage application methods: (i) to the metal union; and (ii) to the sample solution vial.

COMPUTER CODES

Voltage control code for DC voltage scanning (JavaScript)

```
clear()
if(!('StaticIO' in this)) throw "Please open the StaticIO instrument";
if(!('Wavegen' in this) || !('Scope' in this)) throw "Please open a Scope
and a Wavegen instrument";
print("Running StaticIO script");
StaticIO.Channel0.Mode.text = "IOs";
StaticIO.Channel0.DIO7.Mode.text = "Button";
StaticIO.Channel0.DIO7.text = "1";
wait(1); // Unit: s
StaticIO.Channel0.DIO7.text = "0";

Wavegen.run();
var Hz= 0
for(var V = 0.11; V <= 0.32; V = V +0.05){
Wavegen.Channel1.State.text = "Independent"
Wavegen.Channel1.Mode.text = "Simple";
Wavegen.Channel1.Simple.Type.text = "Square";
Wavegen.Channel1.Simple.Frequency.value = Hz; // Unit: Hz
Wavegen.Channel1.Simple.Amplitude.value = V; // Unit: V
Wavegen.Channel1.Simple.Offset.value = V; // Unit: V
Wavegen.Channel1.Simple.Symmetry.value = 50; // Unit: %
Wavegen.Channel1.Simple.Phase.value = 0;
print("start",V,"V",Hz,"Hz");
wait(30);
}
Wavegen.Channel1.Mode.text = "Simple";
Wavegen.Channel1.Simple.Type.text = "DC";
Wavegen.Channel1.Simple.Offset.value = 0;
wait(10);
Wavegen.stop();
print("finish");
```

Stepwise AC voltage frequency control for experimental setup (JavaScript)

```
clear()
if(!('StaticIO' in this)) throw "Please open the StaticIO instrument";
if(!('Wavegen' in this) || !('Scope' in this)) throw "Please open a Scope
and a Wavegen instrument";
print("Running StaticIO script");
StaticIO.Channel0.Mode.text = "IOs";
StaticIO.Channel0.DIO7.Mode.text = "Button";
StaticIO.Channel0.DIO7.text = "1";
wait(1); // Unit: s
StaticIO.Channel0.DIO7.text = "0";

var ampV= 2
var offV= 3

Wavegen.run();
Wavegen.Channel1.Mode.text = "Simple";
Wavegen.Channel1.Simple.Type.text = "DC";
Wavegen.Channel1.Simple.Offset.value = 5;
wait(30);
Wavegen.Channel1.State.text = "Independent"
Wavegen.Channel1.Mode.text = "Simple";
Wavegen.Channel1.Simple.Type.text = "square";
Wavegen.Channel1.Simple.Frequency.value = 1000; // Unit: Hz
Wavegen.Channel1.Simple.Amplitude.value = ampV; // Unit: V
Wavegen.Channel1.Simple.Offset.value = offV; // Unit: V
Wavegen.Channel1.Simple.Symmetry.value = 50; // Unit: %
Wavegen.Channel1.Simple.Phase.value = 0;
print("start",ampV,"V",offV,"V",1000,"Hz");
wait(30);
Wavegen.Channel1.State.text = "Independent"
Wavegen.Channel1.Mode.text = "Simple";
Wavegen.Channel1.Simple.Type.text = "square";
Wavegen.Channel1.Simple.Frequency.value = 5000; // Unit: Hz
Wavegen.Channel1.Simple.Amplitude.value = ampV; // Unit: V
Wavegen.Channel1.Simple.Offset.value = offV; // Unit: V
Wavegen.Channel1.Simple.Symmetry.value = 50; // Unit: %
Wavegen.Channel1.Simple.Phase.value = 0;
print("start",ampV,"V",offV,"V",5000,"Hz");
wait(30);
Wavegen.Channel1.State.text = "Independent"
Wavegen.Channel1.Mode.text = "Simple";
Wavegen.Channel1.Simple.Type.text = "square";
Wavegen.Channel1.Simple.Frequency.value = 10000; // Unit: Hz
Wavegen.Channel1.Simple.Amplitude.value = ampV; // Unit: V
Wavegen.Channel1.Simple.Offset.value = offV; // Unit: V
Wavegen.Channel1.Simple.Symmetry.value = 50; // Unit: %
Wavegen.Channel1.Simple.Phase.value = 0;
print("start",ampV,"V",offV,"V",10000,"Hz");
wait(30);
Wavegen.Channel1.State.text = "Independent"
Wavegen.Channel1.Mode.text = "Simple";
```

```
Wavegen.Channel1.Simple.Type.text = "square";
Wavegen.Channel1.Simple.Frequency.value = 15000; // Unit: Hz
Wavegen.Channel1.Simple.Amplitude.value = ampV; // Unit: V
Wavegen.Channel1.Simple.Offset.value = offV; // Unit: V
Wavegen.Channel1.Simple.Symmetry.value = 50; // Unit: %
Wavegen.Channel1.Simple.Phase.value = 0;
print("start",ampV,"V",offV,"V",15000,"Hz");
wait(30);
Wavegen.Channel1.State.text = "Independent"
Wavegen.Channel1.Mode.text = "Simple";
Wavegen.Channel1.Simple.Type.text = "square";
Wavegen.Channel1.Simple.Frequency.value = 20000; // Unit: Hz
Wavegen.Channel1.Simple.Amplitude.value = ampV; // Unit: V
Wavegen.Channel1.Simple.Offset.value = offV; // Unit: V
Wavegen.Channel1.Simple.Symmetry.value = 50; // Unit: %
Wavegen.Channel1.Simple.Phase.value = 0;
print("start",ampV,"V",offV,"V",20000,"Hz");
wait(30);
Wavegen.Channel1.State.text = "Independent"
Wavegen.Channel1.Mode.text = "Simple";
Wavegen.Channel1.Simple.Type.text = "square";
Wavegen.Channel1.Simple.Frequency.value = 25000; // Unit: Hz
Wavegen.Channel1.Simple.Amplitude.value = ampV; // Unit: V
Wavegen.Channel1.Simple.Offset.value = offV; // Unit: V
Wavegen.Channel1.Simple.Symmetry.value = 50; // Unit: %
Wavegen.Channel1.Simple.Phase.value = 0;
print("start",ampV,"V",offV,"V",25000,"Hz");
wait(30);
Wavegen.Channel1.Mode.text = "Simple";
Wavegen.Channel1.Simple.Type.text = "DC";
Wavegen.Channel1.Simple.Offset.value = 0;
wait(10);
Wavegen.stop();
print("finish");
```

Stepwise AC voltage duty cycle control for experimental setup (JavaScript)

```
clear()
if(!('StaticIO' in this)) throw "Please open the StaticIO instrument";
if(!('Wavegen' in this) || !('Scope' in this)) throw "Please open a Scope
and a Wavegen instrument";
print("Running StaticIO script");
StaticIO.Channel0.Mode.text = "IOs";
StaticIO.Channel0.DIO7.Mode.text = "Button";
StaticIO.Channel0.DIO7.text = "1";
wait(1); // Unit: s
StaticIO.Channel0.DIO7.text = "0";

Wavegen.run();
Wavegen.Channel1.State.text = "Independent"
Wavegen.Channel1.Mode.text = "Simple";
Wavegen.Channel1.Simple.Type.text = "square";
Wavegen.Channel1.Simple.Frequency.value = 20000; // Unit: Hz
Wavegen.Channel1.Simple.Amplitude.value = ampV; // Unit: V
Wavegen.Channel1.Simple.Offset.value = offV; // Unit: V
Wavegen.Channel1.Simple.Symmetry.value = 0; // Unit: %
Wavegen.Channel1.Simple.Phase.value = 0; // Unit: ?
print("start",ampV,"V",offV,"V",20000,"Hz");
wait(20);
Wavegen.Channel1.State.text = "Independent"
Wavegen.Channel1.Mode.text = "Simple";
Wavegen.Channel1.Simple.Type.text = "square";
Wavegen.Channel1.Simple.Frequency.value = 20000; // Unit: Hz
Wavegen.Channel1.Simple.Amplitude.value = ampV; // Unit: V
Wavegen.Channel1.Simple.Offset.value = offV; // Unit: V
Wavegen.Channel1.Simple.Symmetry.value = 10; // Unit: %
Wavegen.Channel1.Simple.Phase.value = 0; // Unit: ?
print("start",ampV,"V",offV,"V",20000,"Hz");
wait(20);
Wavegen.Channel1.State.text = "Independent"
Wavegen.Channel1.Mode.text = "Simple";
Wavegen.Channel1.Simple.Type.text = "square";
Wavegen.Channel1.Simple.Frequency.value = 20000; // Unit: Hz
Wavegen.Channel1.Simple.Amplitude.value = ampV; // Unit: V
Wavegen.Channel1.Simple.Offset.value = offV; // Unit: V
Wavegen.Channel1.Simple.Symmetry.value = 20; // Unit: %
Wavegen.Channel1.Simple.Phase.value = 0; // Unit: ?
print("start",ampV,"V",offV,"V",20000,"Hz");
wait(20);
Wavegen.Channel1.State.text = "Independent"
Wavegen.Channel1.Mode.text = "Simple";
Wavegen.Channel1.Simple.Type.text = "square";
Wavegen.Channel1.Simple.Frequency.value = 20000; // Unit: Hz
Wavegen.Channel1.Simple.Amplitude.value = ampV; // Unit: V
Wavegen.Channel1.Simple.Offset.value = offV; // Unit: V
Wavegen.Channel1.Simple.Symmetry.value = 30; // Unit: %
Wavegen.Channel1.Simple.Phase.value = 0; // Unit: ?
print("start",ampV,"V",offV,"V",20000,"Hz");
```

```
wait(20);
Wavegen.Channel1.State.text = "Independent"
Wavegen.Channel1.Mode.text = "Simple";
Wavegen.Channel1.Simple.Type.text = "square";
Wavegen.Channel1.Simple.Frequency.value = 20000; // Unit: Hz
Wavegen.Channel1.Simple.Amplitude.value = ampV; // Unit: V
Wavegen.Channel1.Simple.Offset.value = offV; // Unit: V
Wavegen.Channel1.Simple.Symmetry.value = 40; // Unit: %
Wavegen.Channel1.Simple.Phase.value = 0; // Unit: ?
print("start",ampV,"V",offV,"V",20000,"Hz");
wait(20);
Wavegen.Channel1.State.text = "Independent"
Wavegen.Channel1.Mode.text = "Simple";
Wavegen.Channel1.Simple.Type.text = "square";
Wavegen.Channel1.Simple.Frequency.value = 20000; // Unit: Hz
Wavegen.Channel1.Simple.Amplitude.value = ampV; // Unit: V
Wavegen.Channel1.Simple.Offset.value = offV; // Unit: V
Wavegen.Channel1.Simple.Symmetry.value = 50; // Unit: %
Wavegen.Channel1.Simple.Phase.value = 0; // Unit: ?
print("start",ampV,"V",offV,"V",20000,"Hz");
wait(20);
Wavegen.Channel1.State.text = "Independent"
Wavegen.Channel1.Mode.text = "Simple";
Wavegen.Channel1.Simple.Type.text = "square";
Wavegen.Channel1.Simple.Frequency.value = 20000; // Unit: Hz
Wavegen.Channel1.Simple.Amplitude.value = ampV; // Unit: V
Wavegen.Channel1.Simple.Offset.value = offV; // Unit: V
Wavegen.Channel1.Simple.Symmetry.value = 60; // Unit: %
Wavegen.Channel1.Simple.Phase.value = 0; // Unit: ?
print("start",ampV,"V",offV,"V",20000,"Hz");
wait(20);
Wavegen.Channel1.State.text = "Independent"
Wavegen.Channel1.Mode.text = "Simple";
Wavegen.Channel1.Simple.Type.text = "square";
Wavegen.Channel1.Simple.Frequency.value = 20000; // Unit: Hz
Wavegen.Channel1.Simple.Amplitude.value = ampV; // Unit: V
Wavegen.Channel1.Simple.Offset.value = offV; // Unit: V
Wavegen.Channel1.Simple.Symmetry.value = 70; // Unit: %
Wavegen.Channel1.Simple.Phase.value = 0; // Unit: ?
print("start",ampV,"V",offV,"V",20000,"Hz");
wait(20);
Wavegen.Channel1.State.text = "Independent"
Wavegen.Channel1.Mode.text = "Simple";
Wavegen.Channel1.Simple.Type.text = "square";
Wavegen.Channel1.Simple.Frequency.value = 20000; // Unit: Hz
Wavegen.Channel1.Simple.Amplitude.value = ampV; // Unit: V
Wavegen.Channel1.Simple.Offset.value = offV; // Unit: V
Wavegen.Channel1.Simple.Symmetry.value = 80; // Unit: %
Wavegen.Channel1.Simple.Phase.value = 0; // Unit: ?
print("start",ampV,"V",offV,"V",20000,"Hz");
wait(20);
Wavegen.Channel1.State.text = "Independent"
Wavegen.Channel1.Mode.text = "Simple";
```

```
Wavegen.Channel1.Simple.Type.text = "square";
Wavegen.Channel1.Simple.Frequency.value = 20000; // Unit: Hz
Wavegen.Channel1.Simple.Amplitude.value = ampV; // Unit: V
Wavegen.Channel1.Simple.Offset.value = offV; // Unit: V
Wavegen.Channel1.Simple.Symmetry.value = 90; // Unit: %
Wavegen.Channel1.Simple.Phase.value = 0; // Unit: ?
print("start",ampV,"V",offV,"V",20000,"Hz");
wait(20);
Wavegen.Channel1.State.text = "Independent"
Wavegen.Channel1.Mode.text = "Simple";
Wavegen.Channel1.Simple.Type.text = "square";
Wavegen.Channel1.Simple.Frequency.value = 20000; // Unit: Hz
Wavegen.Channel1.Simple.Amplitude.value = ampV; // Unit: V
Wavegen.Channel1.Simple.Offset.value = offV; // Unit: V
Wavegen.Channel1.Simple.Symmetry.value = 100; // Unit: %
Wavegen.Channel1.Simple.Phase.value = 0; // Unit: ?
print("start",ampV,"V",offV,"V",20000,"Hz");
wait(20);
Wavegen.Channel1.Mode.text = "Simple";
Wavegen.Channel1.Simple.Type.text = "DC";
Wavegen.Channel1.Simple.Offset.value = 0;
wait(10);
Wavegen.stop();
print("finish");
```
